# Oral and oropharyngeal cancer: Knowledge, attitude and practices among medical and dental practitioners

**DOI:** 10.1002/cnr2.1349

**Published:** 2021-03-03

**Authors:** Latifa Yousef Algudaibi, Shahad AlMeaigel, Nouf AlQahtani, Naila A. Shaheen, Ali Aboalela

**Affiliations:** ^1^ College of Dentistry King Saud bin Abdulaziz University for Health Sciences Riyadh Kingdom of Saudi Arabia; ^2^ King Abdullah International Medical Research Center Riyadh Kingdom of Saudi Arabia; ^3^ National Guard Health Affairs Riyadh Kingdom of Saudi Arabia; ^4^ King Saud Bin‐Abdulaziz University for Health Sciences Riyadh Kingdom of Saudi Arabia

**Keywords:** attitude, dental practitioners, knowledge, medical practitioners, oral cancer, oropharyngeal cancer, practice

## Abstract

**Background:**

Oral and oropharyngeal cancer are significant health problems. They are both life‐threatening conditions usually diagnosed at an advanced stage causing survival rates to decline.

**Aim:**

To assess and compare practices, knowledge and attitude regarding oral and oropharyngeal cancer between dental and medical practitioners.

**Methods:**

A cross‐sectional study was conducted to assess knowledge, attitude and practices of oral and oropharyngeal cancer among dental and medical practitioners at King Abdulaziz Medical City, Riyadh, Saudi Arabia. 360 participants were included in the study using a convenient sampling technique. Participants were approached in their clinics and printed self‐administered questionnaire were handed over to them after signing a written consent form. Frequency distribution and Chi‐Square test were used for the statistical analysis and the level of significance was set at *P* value of .05 or less.

**Results:**

A total of 174 participants responded. Assessment of oral and oropharyngeal cancer knowledge between dental practitioners and medical practitioners showed comparable results. Regarding practices, a significant difference was seen between medical practitioners and dental practitioners in determining the duration of intra‐oral ulcer to consider urgent referral (*P* = .006) and in number of referrals made in relation to suspicious oral lesions (*P* = .002). Moreover, a significant difference (*P* = .006) was seen between medical practitioners and dental practitioners in determining the duration of intra‐oral ulcer to consider urgent referral.

**Conclusion:**

Medical and dental practitioners showed areas of differences in practice, attitude and knowledge of oral and oropharyngeal cancer that when addressed would lead to improved survival rates.

## 1 INTRODUCTION

Oral and Oropharyngeal Cancers (OC/OPC) are both considered significant health problems. When OC/OPC are grouped together they have ranked the fifteenth most common cancers worldwide.^1^ Despite the oral cavity being an accessible site for self and professional examinations and in spite of better understanding and development of new therapeutic interventions, OC/OPC continues to carry a poor survival rate due to late diagnosis. The overall survival rate for OC/OPC is 65%, however, the majority of OC/OPC are diagnosed at an advanced stage having a survival rate of 39%.^2^, ^3^, ^4^ Screening and early detection can lead to a reduction in mortality rate of OC/OPC as in other cancers with well‐developed screening protocols, like breast, lung and colorectal cancers.^5^, ^6^, ^7^, ^8^ Oral cancer refers to any cancerous tissue inside the mouth involving the front two‐thirds of the tongue, floor of the mouth, buccal mucosa, gingiva, lips, retromolar trigone and hard palate. Oropharyngeal Cancers involve the base of tongue, soft palate, tonsils and posterior pharyngeal wall. Most of OC/OPC lesions are squamous cell carcinoma (SSC).^3^ Tumours may arise as a primary lesion in the oral cavity or a metastatic tumour arising from a distant site. Risk factors include smoking, chewing habits (including Areca nut, Shamma/tobacco chewing, Qat, and Toombac), sun exposure, and human papilloma virus (HPV) 16 and 18.^9^, ^10^, ^11^, ^12^, ^13^, ^14^, ^15^, ^16^, ^17^ Globally OC is more common in males but cultural habits have shown to play a role in some regions of the world. An example is the acceptance of women to use shamma (form of smokeless tobacco) in the south western region of Saudi Arabia leading to a higher rate of OC in females in that region with the sites most affected being the gingiva and alveolus (in direct contact with the shamma), whereas the tongue and lips are the most affected sites in other areas of the world.^9^, ^10^, ^11^, ^12^, ^13^, ^14^, ^15^, ^16^, ^17^, ^18^ Cancer patients would benefit from early diagnosis and detection of lesions, with immediate referral to specialist care centres. Early diagnosis and referral will ultimately improve survival rates, reduce morbidity and lead to better treatment outcomes.^4^, ^19^

Dental practitioners (DP) and medical practitioners (MP) are all part of the health care profession but it is likely that the nature of practice would differ between these two groups.^4^, ^20^, ^21^ As far as the authors knowledge, this has been looked at only in the UK, and no other published data comparing the role of medical and dental health care providers with regards to detection of OC/OPC was found.^20^, ^21^ The aim of this study was to assess OC/OCP knowledge, attitude and practices among medical and dental practitioners.

## 2 METHODS

A cross‐sectional study was conducted in order to assess knowledge, attitude and practices of OC/OPC among DP and MP at King Abdulaziz Medical City (KAMC), Riyadh, Saudi Arabia. KAMC has more than 1500 beds and comprised of multiple campuses providing primary, secondary and tertiary care for national guard military and hospital employees and their families. The study inclusion criteria included dentists, dental interns, general medical practitioners, medical interns, family medicine specialists and otorhinolaryngologists who are registered and practicing health care workers. Dental students, dental assistants and dental hygienists are excluded from the study. The study sample could be representative of health care providers in Saudi Arabia as it was performed in a major hospital and all associated primary care centres. An ethics statement was conducted in full accordance with the World Medical Association Declaration of Helsinki. It was independently reviewed and approved by the ethics committee at King Abdullah International Medical Research Centre (KAIMRC), (IRBC/0512/18) study number (SP18/011/R). Written consent form was considered as an agreement for participation in the study and data were collected using a hard copy questionnaire developed by Macpherson et al.^21^ The questionnaire composed of 6 domains: demographics, knowledge of OC/OPC, pattern of referral, preventive role, training needs and examination habits with a total of 37 questions. Knowledge was defined if the participant selected the right option for the prevalence of oral cancer, risk factors, sites and predisposing oral condition. Attitude was assessed by questions about the participants' belief of having a role in several aspects including: prevention, participants' confidence in detecting any premalignant\malignant lesion and pattern of referral of required cases. Practices were based on questions involving routine examination of sign and symptoms, factors influencing decision to undertake examination, sites to focus on during examination, predisposing oral conditions and barriers precluding routine examinations. The questionnaire was estimated to take between 7 to 10 minutes in order to be completed. Participants were selected using convenience sampling technique. The participants were approached in their clinics and after that a brief introduction of the current study was provided. Participants who agreed to participate were handed over the printed self‐administered questionnaire to be filled on the spot. The participants' personal information was anonymously treated for privacy and confidentiality. The data collection process started on 1/1/2019 until 30/4/2019. Statistical analysis was completed by using SAS version 9.4 (SAS Institute, Cary, NC, USA). Frequency and percentages were used to display categorical variables. Chi‐square test was used to test for the presence of association between categorical variables. Level of significance was set at .05.

## 3 RESULTS

Questionnaires were distributed among DP and MP; 174, out of 360, completed questionnaires were received representing 48% response rate. The response rate was higher among the DP (56.9%) of the completed questionnaires compared to the MP (43%).

## 4 DEMOGRAPHIC CHARACTERISTICS

More than half of the respondents were male (64%) while (36%) were female. Half of the DP (50%) had been qualified for less than 5 years, 16% between 6 to 10 years, 6.1% between 11 to 15, 12.1% between 16 to 20 and 16.2% had been qualified for more than 20 years. Similarly, for the MP, about half of (48%) graduated within the last 5 years, 9% between 6 to 10 years, 9% between 11 to 15 years, 11% between 16 to 20 years, and 23% had more than 20 years experience. (Table 1).

**TABLE 1:** Demographic characteristic

Variables	Levels	Study groups		Total
		MP n (%) N = 75	DP n (%) N = 99	
Gender	Male	45 (60)	66 (66.7)	111
	Female	30 (40)	33 (33.3)	63
Nationality	Saudi	60 (80)	89 (89.9)	149
	Non‐Saudi	15 (20)	10 (10.1)	25
Job rank	Intern	25 (33)	35 (35.4)	60
	General	2 (2.7)	9 (9.1)	11
	Resident	13 (17.3)	17 (17.2)	30
	Registrar	11 (14.7)	1 (1)	12
	Specialist	2 (2.7)	3 (3)	5
	Assistant consultant	2 (2.7)	9 (9.1)	11
	Consultant	20 (26.7)	25 (25.3)	45
Years after graduation	0–5 y	36 (48)	49 (49.5)	85
	6–10 y	7 (9.3)	16 (16.2)	23
	11–15 y	7 (9.3)	6 (6.1)	13
	16–20 y	8 (10.7)	12 (12.1)	20
	More than 20 y	17 (22.7)	16 (16.2)	33
Level of post‐graduation	Certificate	8 (10.7)	12 (12.1)	20
	Board	32 (42.7)	30 (30.3)	62
	Master	8 (10.7)	20 (20.2)	28
	PhD	6 (8)	3 (3)	9
	Other	4 (5.3)	3 (3)	7

Abbreviations: DP, dental practitioners; MP, medical practitioners.

### 4.1 Knowledge

#### 4.1.1 OC/OPC prevalence and risk factors

Around 18% of the DP and 25% of the MP estimated the number of new cases of OC diagnosed each year per 100 000 to be less than 50. The largest proportion of both DP (36%) and MP (35%) estimated that the number of new cases is between 51 and 100. While more DP (20%) believed that the number of new OC cases is between 151 to 200 compared to MP (8%). Only 9% of DP and 7% of MP estimated that the number of new cases is more than 200. In relation to OPC, one third (33%) of MP and 26% of DP believed the number of new OPC cases diagnosed each year per 100 000 to be less than 50. Similarly, 31% of MP and 29% of DP estimated that the number of new OPC cases is between 51 and 100. While 20% of the DP believed that the number of new OPC cases is more than 200, only 5% of the MP believed the number to be above 200.

Table 2 shows the respondents perceptions of the perceived importance of OC risk factors. A significant difference between MP and DP was observed in the perception of bacterial infection as an etiology of OC (*P* = .04). In addition, a significant difference between MP and DP was noticed in the perception of sun exposure as an etiology of OC (*P* = .05). No significant difference between MP and DP was observed in perception of age, alcohol, smoking, trauma, HPV, fungal infection, and family history as an etiology of OC.

**TABLE 2:** Respondents perceptions of perceived importance of OC risk factors

	Not aware	Not important	Important	Very important	
Age n (%)					.31
MP	2 (2.7%)	10 (13.3%)	39 (52%)	24 (32%)	
DP	7 (7.1%)	20 (20.2%)	46 (46.5%)	26 (26.3%)	
Alcohol n (%)					.79
MP	2 (2.7%)	4 (5.3%)	20 (26.7%)	49 (65.3%)	
DP	3 (3%)	6 (6.1%)	20 (20.2%)	70 (70.1%)	
Bacterial Infection n (%)					**.04** *
MP	3 (4%)	40 (53.3%)	24 (32%)	8 (10.7%)	
DP	7 (7.1%)	42 (42.4%)	47 (47.5%)	3 (3%)	
Smoking n (%)					0.46
MP	0 (0%)	1 (1.3%)	8 (10.7%)	66 (88%)	
DP	0 (0%)	0 (0%)	13 (13.1%)	86 (86.9%)	
Trauma n (%)					.25
MP	2 (2.7%)	49 (65.3%)	21 (28%)	3 (4%)	
DP	5 (5.1%)	50 (50.5%)	37 (37.4%)	7 (7.1%)	
HPV n (%)					.29
MP	3 (4%)	20 (26.7%)	20 (26.7%)	32 (42.7%)	
DP	5 (5.1%)	15 (15.2%)	33 (33.3%)	46 (46.5%)	
Sun exposure n (%)					**.05** *
MP	2 (2.7%)	31 (41.3%)	23 (30.7%)	19 (25.3%)	
DP	5 (5.1%)	22 (22.2%)	43 (43.4%)	29 (29.3%)	
Fungal infection n (%)					.57
MP	1 (1.3%)	41 (54.7%)	27 (36%)	6 (8%)	
DP	4 (4%)	46 (46.5%)	41 (41.4%)	8 (8.1%)	
Family History n (%)					.16
MP	0 (0%)	8 (10.7%)	28 (37.3%)	39 (52%)	
DP	3 (3%)	4 (4%)	36 (36.4%)	56 (56.6%)	

Abbreviations: DP, dental practitioners, HPV, Human papilloma virus; MP, medical practitioners; OC, oral cancer.

* Significant at level .05.

Table 3 displays the respondents perceptions of perceived importance of OPC risk factors. A significant difference between MP and DP was observed only in perception of trauma as an etiology of OPC (*P* = .05). No significant difference between MP and DP was seen in perception of age, alcohol, smoking, trauma, HPV, sun exposure, fungal infection, and family history as an etiology of OPC.

**TABLE 3:** Respondents perceptions of perceived importance of OPC risk factors

	Not aware	Not important	Important	Very important	
Age n (%)					.2
MP	1 (1.3%)	10 (13.3%)	38 (50.7%)	26 (34.7%)	
DP	6 (6.1%)	20 (20.2%)	40 (40.4%)	33 (33.3%)	
Alcohol n (%)					.46
MP	1 (1.3%)	6 (8%)	17 (22.7%)	51 (68%)	
DP	1 (1%)	3 (3%)	20 (20.2%)	75 (75.8%)	
Bacterial infection n (%)					.11
MP	1 (1.3%)	34 (45.3%)	29 (38.7%)	11 (14.7%)	
DP	6 (6.1%)	39 (39.4%)	47 (47.5%)	7 (7.1%)	
Smoking n (%)					.31
MP	1 (1.3%)	0 (0%)	12 (16%)	62 (82.7%)	
DP	0 (0%)	0 (0%)	11 (11.1%)	88 (88.9%)	
Trauma n (%)					**.05** *
MP	2 (2.7%)	55 (73.3%)	12 (16%)	6 (8%)	
DP	5 (5.1%)	52 (52.5%)	29 (29.3%)	13 (13.1%)	
HPV n (%)					.48
MP	4 (5.3%)	17 (22.7%)	25 (33.3%)	29 (38.7%)	
DP	3 (3%)	16 (16.2%)	32 (32.3%)	48 (48.5%)	
Sun exposure n (%)					.3
MP	1 (1.3%)	48 (64%)	19 (25.3%)	7 (9.3%)	
DP	5 (5.1%)	52 (52.2%)	28 (28.3%)	14 (14.1%)	
Fungal infection n (%)					.88
MP	2 (2.7%)	42 (56%)	26 (34.7%)	5 (6.7%)	
DP	4 (4%)	54 (54.5%)	32 (32.3%)	9 (9.1%)	
Family history n (%)					.23
MP	0 (0%)	7 (9.3%)	27 (36%)	41 (54.7%)	
DP	3 (3%)	4 (4%)	35 (35.4%)	57 (57.6%)	

Abbreviations: DP, dental practitioners; HPV, human papilloma virus; MP, medical practitioners; OPC, oropharyngeal cancer.

* Significant at level .05.

### 4.2 Practices

#### 4.2.1 Examination habits

Significantly higher proportion of the MP (47%) would never examine the patient in the course of the initial examination of a patient greater than 16 years of age for signs of OC/OPC when compared to DP (17%). Most of the participants considered pre‐existing lesion, alcohol and smoking as factors that would influence their decision to undertake an examination for OC/OPC screening.

Lateral borders of the tongue (74%), floor of the mouth (65.5%), pharyngeal wall (60%) and lips (62%) were the sites that the participants mostly focus on when they examined the oral cavity for OC/OPC. Only 47% considered soft palate important to be examined. More DP would consider lateral tongue (83%) and floor of the mouth (79%) examination highly important when compared to MP (63%, 48%), respectively. On the other hand, MP (55%) would consider gingival examination highly important more than the DP (25%).

Lack of training and lack of time were the main barriers to routinely undertake OC/OPC examinations. More MP (53%) perceived lack of time as a very important barrier to OC/OPC examinations when compared to DP (36%).

Leukoplakia (65%), erythroplakia (56%) and smoker's keratosis (56%) were considered to be very important predisposing conditions. In which, about 69% of the DP considered erythroplakia to be very important predisposing condition while only 39% of the MP considered it to be very important. Geographic tongue is considered as an important predisposing condition by MP (43%) more than DP (29%). Similarly, smoker's keratosis considered to be very important by MP (68%), while less DP (47%) perceived it to be very important. In regards to the recall of patients with predisposing oral conditions, DP (73%) would do significantly more than MP (55%).

#### 4.2.2 Pattern of referrals

Table 4 demonstrates the differences in referral pattern between MP and DP. A significant difference between MP and DP was observed in the department of referral (*P* < .001). Majority of the DP (61.7%) would refer a suspicious OC/OPC lesion to an oral medicine department while only 16.9% of the MP would do so. In the other hand, MP has higher proportion of referrals to medical or surgical department like ENT (29.6%), general surgery (9.9%), oral maxillofacial surgery (OMFS) (43.7%) when compared to DP as only 9.9% would refer to ENT, 1.2% to general surgery, and 27.2% to OMFS. Moreover, a significant difference between MP and DP was perceived in number of referrals made in relation to suspicious oral lesions (*P* = .002). In comparison to MP, DP is shown to have a higher number of OC/OPC patient referrals (in all categories). On the other hand, no significant difference between MP and DP was detected in confidence to assess the need for urgent referral.

**TABLE 4:** Differences in pattern of referrals between MP and DP

	MP n (%)	DP n (%)	
Department of referral n (%)			**<.001** *
General surgery	7 (9.9%)	1 (1.2%)	
ENT	21 (29.6%)	8 (9.9%)	
OMFS	31 (43.7%)	22 (27.2%)	
Oral medicine	12 (16.9%)	50 (61.7%)	
Number of referrals made in relation to suspicious oral lesions n (%)			**.002** *
None	52 (69.3%)	39 (39.4%)	
1–5	20 (26.7%)	51 (51.5%)	
6–10	2 (2.7%)	6 (6.1%)	
>10	1 (1.3%)	3 (3%)	
Confident in assessing whether it requires urgent referral n (%)			.46
Very confident	14 (18.7%)	28 (28.3%)	
Confident	31 (41.3%)	40 (40.4%)	
Fairly confident	24 (32%)	25 (25.3%)	
Not Confident	6 (8%)	6 (6.1%)	

Abbreviations: DP: Dental practitioners; ENT: otorhinolaryngologists; MP: Medical practitioners; OMFS: Oral and maxillofacial surgery.

* Significant at level .05.

When the participants were asked about taking biopsies prior to referrals, 64% answered that they never take a biopsy. Regarding competency in knowing which method of referral is appropriate in each situation, majority of both MP and DP where competent 73.3% and 77.8% respectively.

**FIGURE 1:**
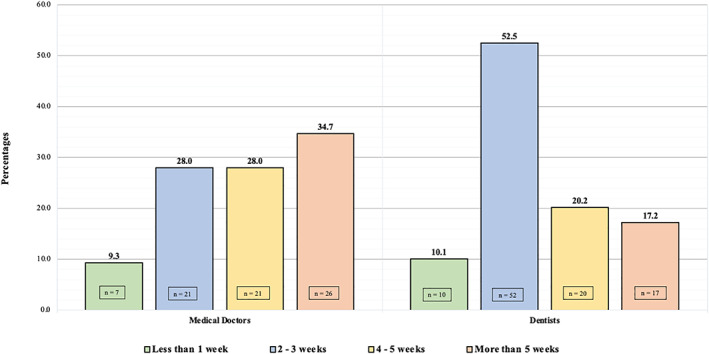
Difference between medical practitioner and dental practitioner in duration for intra‐oral ulcer to consider urgent referral

Figure 1 illustrates the difference between MP and DP in duration for intra‐oral ulcer to consider urgent referral. A significant difference (*P* = .006) was seen between MP and DP in determining the duration of intra‐oral ulcer to consider urgent referral. Majority of DP (52.5%) consider 2 to 3 weeks as maximum duration for intra‐oral ulcer to do urgent referral whereas 28% of MP would refer the patient after 2 to 3 weeks. The largest proportion of MP (34.7%) believes more than 5 weeks as the duration appropriate for urgent referral while 17.5% DP believe so.

### 4.3 Attitude

#### 4.3.1 Practitioner confidence

Half of the participants, both DP and MP, considered themselves to be at least confident in detecting any premalignant\malignant lesion that may be present in the oral cavity, while 10% admitted that they are not confident, and 40% believe that they are fairly confident in detecting any premalignant\malignant lesion.

If they encounter a suspicious OC/OPC lesion, 59% of MP and 52% of DP considered themselves at least confident to discuss the matter with their patients. Around 71% of the participants reported that they are very confident in offering advice in counselling patients on smoking in relation to oral health, while only 33% and 35% admitted that they are very confident in counselling patients regarding alcohol and OC/OPC prevention methods, respectively.

#### 4.3.2 Preventative role

Both participants made enquiries with regards to the patients smoking habits (61% MP, 60% DP) more than alcohol consumption (23% MP, 17% DP). Significantly higher belief of MP (61%) that dental hygienists play a major role in prevention of OC/OPC when compared to DP (34%). About 90% of both participants believe that the dentists play a major role in OC/OPC prevention and a lesser percentage (70%) think that medical doctors play a major role in the prevention.

#### 4.3.3 Training needs

About a third of participants received training in the subject of OC/OPC within the last year, 16% within 1 to 2 years, 22% within 3 to 5 years, 11% within 6 to 10 years ago and 18% last received training more than 11 years ago. Both participants considered courses (50%), journals (40%), and conferences (38%) as the source that contributed to improve their knowledge of OC/OPC. Significantly higher proportion of MP (63%) who have received training in counselling for smoking when compared with DP (37%). Similarly, MP (36%) received more training than DP (21%) regarding alcohol counselling. About 80% of both parties need further information on sources of counselling and support for patients in relation to smoking and alcohol consumption. The majority of the participants would like to have further training in the following areas: detection of oral cancers and pre‐cancer screenings (80.5%), advice on the most appropriate pathway for patient referral (74%), counselling patients on smoking (68%), and counselling patients for cancer prevention (81%). More than half of the participants indicated that the preferred mode of training would be distance learning (58%), and 51% of participants would also like to attend courses and 54% would prefer in‐service training.

## 5 DISCUSSION

The current study was performed to assess knowledge, current practices and training needs of DP and MP with regards to the detection of OC/OPC. Usually, OC/OPC are diagnosed at an advanced stage leading to increased mortality. It is well established that the early diagnosis and management of these patients could increase the survival rate.^22^ In addition, although OC/OPC is known to be diseases of the elderly and mostly with a history of many years of smoking and alcohol consumption, now there is an alarming increase of incidence among young adults.^23^ The increase in incidence has mostly been noted in OPC over the past three decades attributed to HPV.^16^ It is now recognized that awareness and oral cancer screening must involve all patients regardless of age and social history. In the current study, the response rate was higher among DP than MP similar to a study conducted by Macpherson et al. This difference may reflect how both groups perceive their role in OC/OPC prevention, detection and referral.^21^ According to GLOBOCAN estimates of cancer incidence and mortality produced by the International Agency for Research on Cancer, the World Age‐Standardized Rate (ASR) per 100 000 for cancers of the lip and oral cavity in 2018 ranges at its highest at 21.2 in Melanesia and its lowest in Western Africa. For OPC, the world ASR is 1.8 for males and 0.4 for females.^1^ Most of the DP and MP in the current study estimated that the number of new OC/OPC cases are less than 50 or between 51 to 100. While only 5% to 20% of both parties believed that the new OC/OPC cases are more than 200. From these numbers, practitioners might underestimate the incidence of OC/OPC which might affect their screening and referral practice. Smoking and alcohol consumption are the main risk factors for OC.^24^, ^25^, ^26^, ^27^ In our study, participants were knowledgeable about risk factors as the majority of them identified smoking and alcohol as important/very important risk factors. The high level of practitioners' knowledge about OC risk factors coincided with many studies conducted in Saudi Arabia, Australia, and Kuwait.^25^, ^28^, ^29^ An example of regional discrepancies can be seen in Saudi Arabia, where there is an increase in the incidence of OC as in the southern region (Gizan), due to frequent use of special types of smokeless tobacco like Shamma and Qat in the southern region of Saudi Arabia.^30^, ^31^, ^32^ OC/OPC is associated with smoking and alcohol but HPV infections have been found to be an independent risk factor for OPC.^33^, ^34^, ^35^ Nearly a third of the practitioners did not identify HPV as a risk factor for OPC. On the other hand, according to a study conducted on Canadian physicians, only 5% of primary care physicians revealed any doubt regarding the evidence supporting HPV associated head and neck cancer.^36^ Although a significant difference was not found, more DP identified HPV as a risk factor for OPC, when compared to MP. Similarly, dentists showed higher overall HPV related knowledge than dental hygienists in a study examining knowledge of HPV among dentists and dental hygienists attending a regional dental conference in Florida, United States.^37^ This may be due to a more focused training involving the head and neck area.

Practitioners showed areas of differences as both groups, DP and MP, have a good knowledge about OC/OPC risk factors but DP would consider OC/OPC examination and referral more than MP. In the current study, a significantly higher proportion of DP would routinely examine the patients for OC/OPC than MP. That coincides with several studies done in the UK, USA, Italy, and Saudi Arabia.^20^, ^21^, ^38^, ^39^, ^40^ Macpherson et al stated that MP has a general feeling that OC examination is beyond their limits and the DP should do it routinely; this belief was supported also by American dentists and physicians.^21^, ^41^ It is logical that the DP is responsible for OC/OPC screening but there are concerns about how often the patients will visit a dentist. There is evidence that even if the patients were to have oral symptoms, they would visit MP not DP. A British dental survey in 2009 proved that only 58% of the participants visited a dentist in the past 3 years which showed that a large number of the population did not visit their dentists regularly. As a result, dependence on DP to screen patients for OC/OPC might lead to delayed and/or misdiagnosing OC/OPC.^42^ Similar to our findings other studies completed in Saudi Arabia and UK, found that a lack of time and training were reported by both participants as the main barriers to examine patients for OC/OPC.^21^, ^29^ These findings further highlight the need of developing more training opportunities including courses and in‐service training. In addition, by increasing awareness, the hope would be to justify the benefit of time spent when completing these examinations.

High‐risk areas for OC are the posterolateral surfaces of the tongue and the floor of the mouth.^43^ Buccal mucosa is a common OC site in some countries in Asia due to tobacco chewing habit.^44^ In the current study, when the participants were asked about high‐risk sites to focus on during their examination, 74% reported lateral borders of the tongue, 66% floor of the mouth and 54% buccal mucosa. A significantly higher number of DP identified the lateral boarder of the tongue and floor of the mouth as important sites while more MP identified the gingiva as an important site to be examined when compared to DP. This was consistent with a study done in the US; less than 10% of MP and 39% of DP identified the most common site for OC.^41^ These findings indicate that MP who are more likely to see patients first would not be wary of high‐risk sites. In this study, more participants identified leukoplakia (65%) to be a very important predisposing condition when compared to erythroplakia (56%). erythroplakia was significantly identified by DP to be of more risk than MP. Applebaum et al in the US reported that less than 10% of MP and 34% of DP recognized leukoplakia and erythroplakia as the two oral changes associated with OC.^41^ In addition, Carter et al in UK reported that a significantly lower number of MP identified leukoplakia and erythroplakia as the two predisposing conditions for OC.^20^ When we compare our results to the previous studies, our participants identified the two predisposing conditions more than participants in the UK and US but were similar in that DP was able to identify the two conditions more than the MP. Literature reviews^45^, ^46^ reported that the rate of malignancy transformation of erythroplakia (9%‐40%) is much more than leukoplakia (2%‐6%) and at time of biopsy (91%) of erythroplakias were found to be dysplastic to carcinoma in situ when compared to (20%) of leukoplakias at time of biopsy.^47^, ^48^ These figures represent the urge for all health care providers to be able to identify these conditions in their early phases to prevent detect early cancerous lesions.^21^

In our study, DP and MP significantly differ in the referral pattern; MP would mostly refer suspicious lesions to OMFS (41%) or ENT (28%) department while DP would primarily refer to the oral medicine department (51%). Comparable results were reported by Carter et al in UK; he reported that OMFS and oral medicine departments are the two main specialties receiving referrals by both MP and DP although DP referred more to oral medicine departments when compared to MP.^20^ On the other hand, Macpherson et al stated that both MP and DP mostly refer to OMFS departments.^21^ DP in the current study refers more oral cancer cases in comparison to MP. Similarly, Applebaum et al in the US found that the mean number of patients receiving biopsy/referred for diagnosis of suspicious oral lesion in the past 12 months is higher among DP than MP.^41^ The majority of DP urgently referred suspicious intra‐oral ulcers after 2 to 3 weeks while more MP would do so after 5 weeks. Comparable results were found by Macpherson et al; he reported that the majority of DP would consider urgent referrals after 2 to 3 weeks and over half of MP would refer suspicious ulcers after 4 to 5 weeks.^21^ Practitioners should refer oral ulcers if persisting for more than 3 weeks.^43^, ^49^ It was found that DP adheres to the guidelines more than MP but a large proportion of both practitioners were found to be willing to wait more than 3 weeks before considering an urgent referral, which might lead to delayed diagnosis, affecting the survival rate.^21^ This might be due to lack of emphasis on the importance of urgent referral of such cases during undergraduate or training years. No significant difference in practitioners' confidence was observed between DP and MP in the current study while in the study conducted by macpherson et al. DP was more confident in detecting pre‐malignant/malignant oral lesions and assessing the need for urgent referrals.^21^ The level of confidence in detecting any oral premalignant\malignant lesion of both practitioners (50%) in our study is more than what has been reported by Macpherson et al (15% MP, 30% DP).^21^

In the current study, participants assess their patients regarding smoking more than alcohol consumption although they reported high knowledge level in identifying smoking and alcohol as the main OC/OPC risk factors. This might be related to the religious or legal regulations in Saudi Arabia that prohibit alcohol consumption or because they lack the confidence as only 33% of the participants admitted that they were very confident in counselling patients regarding alcohol consumption compared to smoking 71%. This result coincides with an earlier study conducted in Saudi Arabia by Jaber et al; he stated that alcohol consumption is within the least assessed items by practitioners.^29^ Also, the previous results are consistent with many studies conducted in UK, Kuwait and the US.^21^, ^25^, ^50^, ^51^

Both practitioners in the current study agreed that DP have the major role in OC/OPC prevention then MP and lastly community pharmacists. More MP believe that dental hygienists have major role in cancer prevention. This identifies a critical perception, that if changed would have great benefit, as patients tend to see their primary care physician more than their dentist. In addition, the community pharmacist could play a role in detecting oral lesions. Furthermore, dental hygienists are considered as prevention specialists; they might spend more time with the patients and see higher number of patients compared to DP.^37^ As a result, awareness must be raised for all health care providers especially primary care providers with regards to known risk factors, most common sites and early signs for OC/OPC.^21^ In our study, MP received more training than DP regarding smoking and alcohol counselling. Majority of both practitioners need further information and training on sources of counselling patients regarding smoking, alcohol consumption, cancer prevention, detection of oral cancer/ pre‐cancer and patient referral.

Distance learning, courses and in‐service training are the preferred methods of training by our participants. Carter et al in UK similarly reported that the majority of DP and MP needed further training in OC but they preferred information pack rather than courses or meetings.^20^

The limitations of the current study included a low response rate which may have been due to the lengthy questionnaire and practitioners' busy schedules. Second, the study was done in an institution that continually provides training and education in all aspects of cancer and may have affected the results of our study positively.

## 6 CONCLUSION

Knowledge, attitude and training with regards to OC/OPC were all found to be deficient. DP was found to be more knowledgeable about the high OC/OPC risk sites and predisposing factors than MP. In addition, DP perform routine OC/OPC examination and proper referral more than MP. More education and training with regards to OC/OPC examination and referral should be addressed, through systemic educational updates. This would lead to improved patient care and outcomes by leading to early diagnosis and immediate referrals to specialist care.

## CONFLICT OF INTEREST

The author declare that they have no conflict of interest.

## AUTHOR CONTRIBUTIONS

All authors had full access to the data in the study and take responsibility for the integrity of the data and the accuracy of the data analysis. *Conceptualization*, L.A., S.A., N.A.; *Data Curation*, L.A., S.A., N.A.; *Investigation*, L.A., S.A., N.A.; *Methodology*, L.A., S.A., N.A.; *Resources*, L.A., S.A., N.A.; *Writing‐Original Draft*, L.A.; *Writing‐Review & Editing*, L.A., A.A.; *Formal Analysis*, N.A.S.; *Software*, N.A.S.; *Project Administration*, A.A.; *Supervision*, A.A.; *Validation*, A.A.; *Visualization*, A.A.

## ETHICS STATEMENT

An ethics statement was conducted in full accordance with the World Medical Association Declaration of Helsinki. The study was approved by The Institutional Review Board at King Abdullah International Medical Research Center, Riyadh, Saudi Arabia on 19 February; the approval number is IRBC/0512/18.

## CONSENT TO PARTICIPATE

Written consent form was considered as an agreement for participation in the study.

## Data Availability

The data that support the findings of this study are available from the corresponding author upon reasonable request.
